# The Impact of Lifestyle, Diet and Physical Activity on Epigenetic Changes in the Offspring—A Systematic Review

**DOI:** 10.3390/nu13082821

**Published:** 2021-08-17

**Authors:** Louise Rasmussen, Sine Knorr, Christian Skødt Antoniussen, Jens Meldgaard Bruun, Per Glud Ovesen, Jens Fuglsang, Ulla Kampmann

**Affiliations:** 1 Department of Obstetrics and Gynaecology, Aarhus University Hospital, Palle-Juul-Jensens Boulevard 99, 8200 Aarhus N, Denmark; loura7@rm.dk (L.R.); per.ovesen@clin.au.dk (P.G.O.); jens.fuglsang@skejby.rm.dk (J.F.); 2 Steno Diabetes Center Aarhus, Aarhus University Hospital, Hedeager 3, 8200 Aarhus N, Denmark; sine.knorr@clin.au.dk (S.K.); jens.bruun@clin.au.dk (J.M.B.); 3 Department of Public Health, Aarhus University, Batholins Allé 2, 8000 Aarhus C, Denmark; csa@ph.au.dk; 4 Department of Clinical Medicine, Aarhus University, Palle Juul-Jensens Boulevar 82, 8200 Aarhus N, Denmark

**Keywords:** pregnancy, epigenetics, DNA methylation, miRNA, diet, physical activity, healthy lifestyle

## Abstract

Aims: This systematic review examines the association between maternal lifestyle, diet and physical activity, and epigenetic changes in the offspring. Methods: A literature search was conducted using multiple science databases: PubMed, Embase and Cochrane Library, on 10 March 2021. RCT and Cohort studies in English or Scandinavian languages were included. Exposure variables included diet, lifestyle, meal patterns or physical activity. Studies using dietary supplements as exposure variables were excluded. Outcome variables included were DNA methylation, microRNA or histone changes in placenta, cord blood or offspring. Two independent authors screened, read and extracted data from the included papers. The Cochrane risk-of-bias tool for randomized trials (RoB2) and The Critical Appraisal Skills Program (CASP) Cohort Study Checklist were used to assess risk of bias in the included studies. A qualitative approach was employed due to heterogeneity of exposures and results of the studies. Results: 16 studies and 3617 participants were included in the final analysis. The exposure variables included physical activity, carbohydrate, low glycemic index diet, added sugar, fat, Mediterranean diet and pro-inflammatory diet. The outcome variables identified were differences in DNA methylation and microRNA. Most studies described epigenetic changes in either placenta or cord blood. Genes reported to be methylated were GR, HSD2, IGF-2, PLAG1, MEG-3, H19 and RXRA. However, not all studies found epigenetic changes strong enough to pass multiple testing, and the study quality varied. Conclusion: Despite the variable quality of the included studies, the results in this review suggest that there may be an association between the mother’s lifestyle, diet and level of physical activity during pregnancy and epigenetic changes in the offspring.

## 1. Introduction

The prenatal development process is a critical period where variation, overload or deficiency of nutrients can affect the fetus through fetal programming that may have persistent effects on the health of the offspring and induce the risk of disease later in life [1,2]. Parental nutrition and lifestyle have been associated with long-term health consequences of the fetus [2,3], and coinciding with the present increase in unhealthy lifestyle, the risk of developing lifestyle diseases early in life is increasing [4]. Epigenetic variation is a plausible link between maternal lifestyle and later disease susceptibility in the offspring [5].

Genomically imprinted genes may play a critical role in terms of fetal development and disease processes by epigenetic changes [6]. Epigenetic changes can alter gene expression without changing the DNA-sequence and include DNA methylation, histone modification and micro-RNA (miRNA) changes [7].

Changes in DNA methylation is the best-described epigenetic modification and occurs when a methyl group is added to the cytosine at the five position in a pyrimidine ring (5′-C) in a cytosine-phosphate-guanine (CpG) dinucleotide [2,7]. The mechanism of DNA methylation is used by the cells to ‘lock off’ or ‘silence’ genes. Depending on the site of methylation, expression of the gene can be downregulated or promoted [2]. In general, with increased DNA methylation, the activity of the gene will be downregulated [4]. Whether it is advantageous in terms of programming for a gene to be methylated to a greater or lesser degree depends on the specific gene [4]. miRNA are small RNA molecules that can bind to specific transcribed mRNA sequences, and thereby lower their expression [7]. Histone modifications can alter the chromatin structure, which results in either increased or decreased transcriptional activity [7].

A recent systematic review suggests that maternal BMI is associated with some epigenetic changes in the placenta and offspring [7]. Environmental factors, such as maternal lifestyle, including diet and level of physical activity or sedentary time, may also be associated with epigenetic modifications that can alter gene expression. It is therefore relevant to investigate whether genetic alterations in the offspring can be induced by an altered maternal behavior. This systematic review aimed to summarize the current literature on associations between lifestyle, including diet and exercise, and epigenetic markers.

## 2. Materials and Methods

This systematic review was performed according to the PRISMA statement for reporting systematic reviews [8]. The protocol was registered in PROSPERO (registration number: CRD42021242137, available online at https://www.crd.york.ac.uk/PROSPERO). Eligibility was assessed for all studies that reported paternal or maternal lifestyle (dietary patterns, specific dietary components and/or physical activity) prior to and/or during pregnancy associated with epigenetic modifications in placental or offspring tissues and fluids. Only papers published in English or Scandinavian languages were assessed. Due to the multifaceted interventions and exposures in terms of both diet and physical activity and diverse outcomes for all types of epigenetic markers, we chose a descriptive design and not a meta-analysis. The primary outcomes reported are associations between lifestyle (diet, physical activity or both) and DNA methylation, histone modifications or miRNA.

Comprehensive searches in PubMed (which includes the MEDLINE database), Embase and Cochrane Library databases were completed on 10 March 2021. The search strategy was developed in collaboration with an experienced research librarian. Both controlled vocabulary terms and keywords were used. We used a combination of the following terms and synonyms: “Pregnancy”, “Maternal”, “Paternal”, “Infant”, “Periconception”, “miRNA”, “DNA Methylation”, “Epigenetic”, “Epigenomics”, “Diet”, “Nutrients”, “Exercise” and “Physical Activity”. The full search is shown in Appendix A, and in Appendix B, an example of a search block in PubMed is shown.

Covidence [9] was used as tool in the screening process. Two independent authors (U.K. and S.K. or L.R. and C.A.) reviewed the searches, and read and extracted all relevant abstracts. Two independent authors (U.K. and S.K. or L.R. and C.A.) performed the full-text screening and read the included papers. In case of any disagreement, a third reviewer was involved. Two independent reviewers read and discussed the included articles.

Studies that used dietary supplements as exposure variables were excluded as we aimed to assess the effect of a healthy lifestyle, including diet and exercise, instead of assessing the potential epigenetic modifications due to single vitamins, minerals or micronutrients.

### Data Extraction and Quality Assessment

We used a self-developed template (Appendix C) to extract relevant study data. Two independent authors (U.K. and S.K. or L.R. and C.A.) systematically extracted information on publication year, authors, study design, sample size, participants, tissue chosen for epigenetics analysis, exposure/intervention type, tool used for measurements of outcome, handling of potential cofounders such as ethnicity and smoking, characteristics of control and comparison groups (Table 1), as well as outcome measures (DNA methylation, microRNA or histone) and main findings (Table 2). To identify potential consistencies in findings, we systematically sorted results in ‘Specific Genome Approach’ and ‘Genome-Wide Approach’ in Table 1 and sorted results corresponding to exposure (specific macronutrient, diet, physical activity, etc.) in Table 2.

Two independent authors (U.K. and S.K. or L.R. and C.S.A.) assessed the methodological quality of each study using the Revised Cochrane risk-of-bias tool for randomized trials (RoB2) template [10] for randomized controlled trials (RCTs), and the Critical Appraisal Skills Program (CASP) Cohort Study Checklist [11] for cohort studies. Results were described and tabulated.

## 3. Results

### 3.1. Search Results

**Figure 1:**
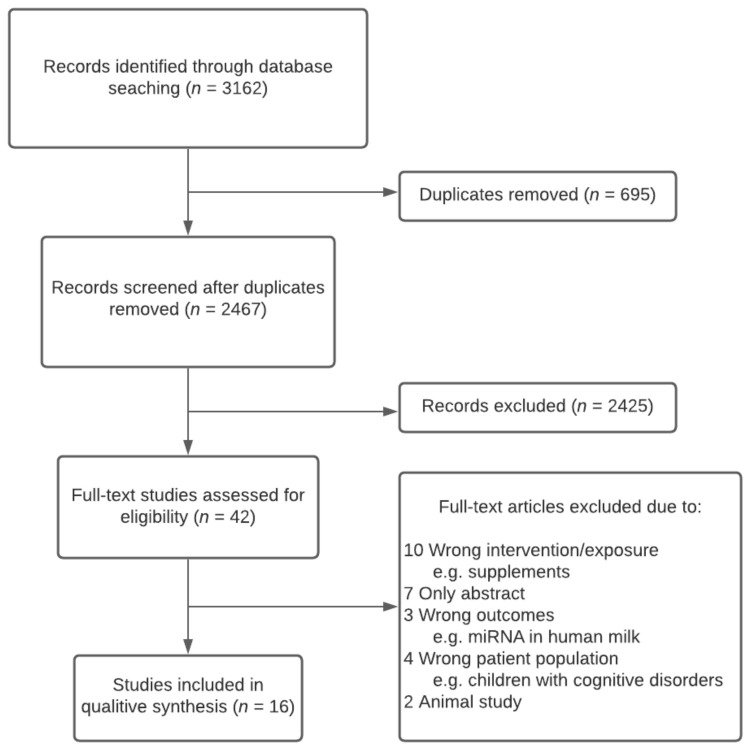
Study flowchart.

A total of 2467 records were identified after the removal of duplicates. After screening the titles and abstracts of the articles, 42 full-text articles were assessed for eligibility. A further 26 were excluded due to wrong intervention, wrong outcomes, wrong patient population, animal study, wrong study design or only abstract available. We identified 16 studies that met the inclusion criteria, and these studies were included in the review. The study selection process is shown in Figure 1.

### 3.2. Summary of Study Characteristics

Among the 16 studies included, 5 studies were RCTs [17,18,19,20,21,26] and 11 studies were cohort studies [3,4,6,12,13,14,16,22,23,24,25]. In total, 3617 mother–offspring pairs were included. Seven studies included participants from the USA [3,4,6], two from the United Kingdom (UK) [14,17], two from Ireland [18,19] and the rest of the studies included participants from China [24], Japan [16], Argentina [20], Denmark [21] and Scotland [13]. Two studies included data from the same RCT that investigated epigenetic changes at birth and at 5 years of age in offspring of mothers on a low glycemic index diet (Geraghty et al. [19] and Geraghty et al. [18], respectively).

We identified no studies focusing on paternal exposures. All RCT studies and most of the cohort studies investigated intervention/exposures during maternal pregnancy. None of the RCT studies examined an intervention in the preconception period or periconceptional period (period from before conception to early pregnancy). The exposure variables were found to vary considerably. Some of the included studies investigated a lifestyle with a combination of diet and physical activity, while others investigated either a specific diet or dietary components. Four studies investigated physical activity as the exposure variable [4,6,17,21], four studies investigated carbohydrate intake [12,14,16,22], four studies investigated glycemic index [17,18,19,24], two studies investigated sugar intake [12,23], two studies investigated Mediterranean diet [21,25], four studies investigated fat intake [12,17,22,27] and three studied other exposures (olive oil [20], unbalanced diet [13] and pro-inflammatory diet [3]). Most studies investigated epigenetic changes in the offspring at birth. However, two studies investigated epigenetic changes later in life: offspring at 5 years [18] and at 40 years of age [13].

We identified no studies that investigated histone changes and only one that study investigated miRNA changes [20]. The remaining 15 studies investigated DNA methylation, of which 7 of the included studies used a genome-wide approach [17,18,19,21,22,23,24] measuring methylation at CpG sites or differentially methylated regions (DMRs), while eight studies investigated DNA methylation at specific genes [3,4,6,12,13,14,16,25]. Seven studies used cord blood [3,6,14,17,19,21,25], five studies used placenta samples [12,20,22,23,24] and the rest of the studies used material from either saliva [18], umbilical cord (UC) tissue [16], blood spots [4] or peripheral blood [13].

The characteristics of the studies sorted by the specific gene approach and genome-wide approach are shown in Table 1. Study outcomes and main results sorted by exposure are depicted in Table 2.

The following acronyms are used: ADD2, adducin 2; DMR, differentially methylated region; CpG, cytosine-phosphate-guanine; FDR, false discovery rate; GDM, gestational diabetes mellitus; HA, high active; HSD2, hydroxysteroid dehydrogenase type 2; IGF2, insulin growth factor 2; LA, low active; LINE, long interspersed nuclear elements; MEG3, maternally expressed 3; MEST, mesoderm-specific transcript; NNAT, neuronatin; PEG, polyethylen glycol; PLAG1, pleomorphic adenoma gene 1; RXRA, retinoid X receptor alpha; SGCE, sarcoglycan epsilon; SNP, single-nucleotide polymorphism; SSTR4, somatostatin receptor 4; ÚTR, 5′ untranslated region.

### 3.3. Study Quality

The methodological quality of the included studies is shown in Table 3 and Table 4. Of the included RCT studies, three of the studies showed a low risk of bias (Table 4), while some concerns in relation to the risk of bias were associated with the two remaining studies. For the included cohort studies, the study quality varied considerably (Table 3), and most of the studies had methodological flaws as several of the studies had not identified and adjusted for all important potential confounding factors, which may have resulted in bias. In one study, it was uncertain whether the authors had adjusted for important confounding factors [3,4,6,16,22,24].

### 3.4. Specific Gene Approach

Eight cohort studies and no RCTs applied a specific gene approach. Seven of the eight studies showed significant differences in methylation levels between groups in at least one gene [4,6,12,13,14,16,25]. Daniels et al. [12] and Godfrey et al. [14] found that lower maternal carbohydrate intake was associated with higher DNA methylation. Thakali et al. [22] also found that carbohydrate intake was significantly associated with methylation of 12 CpGs. However, it was not significantly associated with placental DNA methylation when adjusting for multiple testing. Miyaso et al. [16] also showed that increased carbohydrate intake tended to be related to lower methylation levels. However, the association was not significant (*p* = 0.067).

Further, Daniels et al. [12] and Godfrey et al. [14] also measured the association between protein and fat intake and DNA methylation. None of them found any significant associations. However, Thakali et al. [22] examined maternal saturated fat intake and found that it was significantly associated with placental methylation at 302 of approximately 300,000 CpGs. Drake et al. [13] observed an association between methylation and increased meat intake (*p* = 0.03) in mothers who had an unbalanced diet in pregnancy.

According to caloric intake, Daniels et al. [12] investigated the association between total caloric intake and placenta leptin gene methylation and found a significant association (*p* < 0.05), however after controlling for relevant covariates, the significance disappeared. Miyaso et al. [16] observed an association between a caloric intake of less than 1000 kcal/day and lower methylation levels (*p* = 0.013).

McCullough et al.’s work [3] was the only study investigating the association between a pro-inflammatory diet and methylation levels. They found no evidence of an association between this diet pattern and methylation status at 9 DMRs. However, Gonzalez-Nahm et al. [25] investigated the association between methylation levels of MEG3, IGF2, PLAGL1, H19, MEST, NNAT, PEG3 and SGCE/PEG10 in offspring cord blood and adherence to a Mediterranean diet—a diet reported for its ability to reduce inflammation [25]. The authors found that female offspring of mothers with a low adherence to a Mediterranean diet had greater odds of hypomethylation at the MEG3-IG DMR. After sex-specific adjustment, there was also a significant effect of low Mediterranean diet adherence and the odds of higher methylation in boys at the PLAG1 and H19 DMRs (H19: OR: 4.46, 95% CI: 1.32–15.08; PLAGL1: OR: 3.24, 95% CI: 1.02–10.26). The authors thus indicated a sex-specific impact on infant DNA methylation at specific imprinted DMRs.

Two of the studies, with a specific gene approach, examined the association between physical activity and DNA methylation [4,6]. McCullough et al. [6] investigated the association of non-sedentary time on four imprinted genes: H19, MEG3, SGCE/PEG10 and PLAGL1 DMRs, in cord blood. Non-sedentary time decreased methylation at the PLAGL1 DMR and appeared to be consistent with a threshold effect (*p* = 0.0136). Marshall et al. [4] divided the participants into ‘high active’ participants (average 637.5 min per week of leisure-time physical activity) and ‘low active’ participants (average 59.5 min per week) and also found a significant difference between groups.

Collectively, these studies suggest that there is an association between maternal lifestyle and offspring DNA methylation on various genes.

### 3.5. Genome-Wide Approach

Five RCTs [17,18,19,20,21] and three cohort studies [22,23,24] examined genome-wide methylation levels. Antoun et al. [17] and Jönsson et al. [21] both investigated an intervention of a combination of diet and physical activity on DNA methylation in cord blood. Jönsson et al. [21] studied the influence of a low-fat and low-energy Mediterranean diet combined with physical activity on DNA methylation in pregnant women with obesity. They found that DNA methylation was altered in the intervention group at 379 sites, annotated to 370 genes that are primarily involved in metabolic processes. Antoun et al. [17] investigated a diet with low glycemic index (GI) and a reduced intake of saturated fat combined with physical activity in pregnant women with obesity. Overall, Antoun et al. [17] found no difference in methylation between the intervention group and the control group, but maternal GDM and 1 h glucose concentration following an oral glucose tolerance test (OGTT) were associated with 242, 1, 592 and 17 differentially methylated dmCpG, and these methylation signatures appeared to be attenuated by the dietary and physical activity intervention during pregnancy. Yan et al. [24] investigated the impact of a low glycemic index diet on placental DNA methylation, and in accordance with the study by Antoun et al., Yan et al. found an effect on DNA methylation, specifically on methylation levels of cg17586860 and cg18197392 in the 5′ UTR region of SSTR4. Geraghty et al. [18,19] also investigated the effect of a low glycemic diet during pregnancy. They found that a low glycemic diet during pregnancy induced widespread DNA methylation changes in cord blood, but the association was not strong enough to remain significant after correction for multiple testing [19]. In a later study, Geraghty et al. investigated the genome-wide DNA methylation changes in saliva from offspring of 5 years old born to mothers who participated in the same RCT [18]. No association was found between offspring DNA methylation and the dietary intervention during pregnancy (adjusted *p* < 0.05). Linear regression analysis revealed no significant differentially methylated probes between the two groups using an adjusted *p* > 0.05. However, applying an un-adjusted *p* < 0.05 identified 22,181 CPGs.

Thakali et al. [22] studied the association between genome-wide DNA methylation changes in placenta and maternal fat, carbohydrate, protein and saturated fat intake during pregnancy. Similar to Daniels et al. [12] and Godfrey et al. [14], who used the gene-specific approach, Thakali et al. found that maternal carbohydrate intake during pregnancy was significantly associated with DNA methylation levels. More specifically, they found that maternal carbohydrate, protein and total fat intake were significantly associated with methylation of 12, 14 and 28 CpGs, respectively. However, no maternal diet predictor variable was significantly associated with placental DNA methylation when adjusting for multiple testing. Trumpff et al. [23] focused on one specific type of carbohydrates, namely, added sugar. They observed DNA methylation levels in placenta in 453,144 CpG sites, but no individual CpG achieved significance for altered methylation. However, a secondary analysis revealed that added sugar intake in the third trimester was associated with increased DNA methylation of a cluster of 8 CpGs within the ADD2 gene.

Gomez Ribot et al.’s work [20] was the only study examining methylation of microRNA. They investigated the relative expression of microRNA’s, miR-130a and miR-518d, in placenta of offspring born to mothers with GDM who participated in a RCT investigating the effect of three tablespoons of olive oil as a supplement to their regular diet during pregnancy, as compared to healthy controls. The authors found 518d expression to be increased in the placenta of GDM mothers but reduced in the GDM mothers who received the intervention (*p* = 0.009). No effect was found on the miR-130a expression.

## 4. Discussion

Epigenetic changes during pregnancy could be the link between maternal lifestyle and later risk of chronic diseases, including obesity in the offspring. To the authors’ knowledge, this is the first systematic review to examine the relationship between maternal lifestyle, diet and physical activity during pregnancy, and epigenetic changes in the offspring.

The studies included in this review suggest that diet and physical activity during pregnancy can influence offspring DNA methylation at several DMRs and CpG sites related to cell proliferation, cancer development, obesity, type 2 diabetes and immune and inflammatory responses. However, the studies investigated different exposures and outcomes and showed varying results.

### 4.1. Specific Gene Approach

Although the exposures investigated in the included studies displayed some degree of heterogeneity, all studies, except one, with a gene-specific approach found significant differences in methylation levels between groups. Drake et al. [13] found that offspring of mothers with a high meat/fish and vegetable intake combined with a low bread/potato intake had higher mean methylation at the glucocorticoid receptor (GR) exon 1F, measured in peripheral blood. Methylation was also increased at a specific CpG site at 11β-hydroxysteroid dehydrogenase type 2 (HSD2) region 2 in the offspring of mothers with an increased meat and fish intake. The methylation of the GR-gene is interesting as glucocorticoids play a crucial role in metabolism and tissue development. As the access of glucocorticoids to GR is modulated by HSD2, this gene was also studied. Methylation at the HSD2 decreases the gene expression, and reduced activity of HSD2 is associated with hypertension. The insulin-like growth factor 2 (IGF-2) gene, being an important prenatal growth factor, was also studied. However, no methylation was observed in the IGF-2 gene [13].

Gonzales-Nahm et al. [25] also examined the IGF-2 gene in umbilical cord blood from pregnant women who adhered to a Mediterranean diet during pregnancy, but no methylation changes could be found. However, regarding IGF-2 and exercise, Marshall et al. [4] found in infants blood spots that gene methylation occurred at two CpG sites in the P2 promoter within the IGF2 gene in offspring of physically active mothers. Thus, in offspring of mothers who were in the low active group, there was a significantly higher DNA methylation compared to offspring of mothers in the high active group. Another study in the current review, focusing on the potential effects of maternal physical activity on DNA methylation in cord blood, was performed by McCullough et al. [6], where non-sedentary time decreased methylation at the PLAG1 DMR and was ascribed an association between maternal physical activity and offspring birthweight. The gene is part of a network of genes involved in the control of embryonic growth. Loss of methylation at the PLAG1 DMR during fetal development is also thought to be the causal factor for transient neonatal diabetes mellitus (TNDM) [28]. A gene that was affected by a Mediterranean diet in the study by Gonzalez-Nahm [25] was the tumor suppressor gene MEG-3 [29,30,31], as female infants of mothers with a low adherence to a Mediterranean diet had higher odds of hypo-methylation at the MEG-3 DMR. McCollough et al. [3] also examined the association between methylation changes at MEG-3 in cord blood in offspring of mothers consuming a pro-inflammatory diet during pregnancy, but no association between maternal intake of a pro-inflammatory dietary pattern and methylation of the DMRs could be found. Miyaso et al. [16] focused on another tumor suppressor gene, namely H19 [32]. As the authors state, changes in the methylation of the H19 DMR are related to human health, but little is known about the factors that regulate the methylation of H19 DMR. Miyaso et al. [16] investigated H19 DMR methylation in umbilical cord tissue and found that methylation was significantly reduced in offspring of mothers whose caloric intake was less than 1000 kcal/day during pregnancy. Daniels et al. [12] investigated the associations between maternal diet and placental leptin gene methylation and found that lower levels of leptin methylation were significantly associated with greater intake of carbohydrates. Leptin is a hormone primarily released from the adipose tissue and relates to the amount of adipose tissue in the body. Leptin has several endocrine functions, such as appetite regulation, immune and inflammatory response/function, hematopoiesis, angiogenesis, reproduction and bone formation. Mutations in the leptin gene and its regulatory regions can cause severe obesity and are linked to development of type 2 diabetes [33,34]. Godfrey et al. [14] found that the RXRA gene was affected by carbohydrates, as a higher methylation of RXRA chr9:136355885 in cord blood was found to be associated with lower maternal carbohydrate intake. Fat and protein intake were not associated with DNA methylation changes. The RXRA gene codes for the retinoid X receptors (RXRs), which are nuclear receptors and mediate the biological effects of retinoids by their involvement in retinoic acid-mediated gene activation. The protein encoded by this gene is a member of the steroid and thyroid hormone receptor superfamily of transcriptional regulators and is involved in a range of physiological processes. An altered gene expression of RXRA is involved in the development of many diseases. Active RXRα is among other things required for ocular morphogenesis and the late steps in trophoblast differentiation [35].

### 4.2. Genome-Wide Approach

Among the studies with a genome-wide approach, the results were more varying and similar to the studies with the specific gene approach, and there was a large heterogeneity in terms of exposure, tissues and outcome measurements. Jönsson et al. [21] and Antoun et al. [17], both undertaking RCTs, investigated a diet combined with a physical activity intervention and both found an effect on DNA methylation in cord blood. In a recent meta-analysis [36], the effect of maternal exercise during pregnancy on growth and childhood obesity was examined, and a reduced risk of large for gestational age (LGA) and reduced childhood obesity in offspring from mothers with normal weight was found. Perhaps the epigenetic changes such as those found by Jonsson et al. and Antoun et al. are the explanation for the beneficial effect of physical activity.

Yan et al. [24] focused on glycemia during pregnancy and found that methylation levels of cg17586860 and cg18197392 in the 5′ UTR region of SSTR4 in placental tissue DNA were negatively correlated with changes in carbohydrate intake across gestation. The SSTR4 gene is the somatostatin receptor gene. The hormone somatostatin (SST) affects many sites to inhibit the release of many hormones and other secretory proteins. The SSTR4 is a member of the superfamily of receptors having seven transmembrane segments and is primarily expressed in fetal and adult brain and lungs [37].

Geraghty et al. [19], Geraghty et al. [18] and Thakali et al. [22] found widespread DNA methylation changes, however, none strong enough to pass multiple testing.

The study by Gomez Ribot et al. [20] was the only included in the current review that examined miRNA changes. The authors found that the miRNA 518d expression was increased in the placenta of GDM mothers but reduced in those GDM mothers receiving an intervention of olive oil during pregnancy. In a recent systematic review [7], examining epigenetic changes associated with maternal BMI and gestational weight gain, an association between maternal BMI and changes in miRNA expression was observed in several studies, and it is thus plausible that lifestyle, diet and physical activity in general may have an influence on miRNA.

### 4.3. Strengths and Limitations

Although there are indications of an epigenetic effect-modification of diet and exercise in the current review, there are several limitations.

First, the exposures are heterogenic, including different dietary patterns, dietary components and physical activity levels. Moreover, only two different epigenetic measures were included (microRNA expression and DNA methylation). Thus, the influence of dietary and lifestyle exposures was only investigated on microRNA expression and DNA methylation. Although these might be the most studied epigenetic changes, histone modifications may be more responsive to environmental stimuli and histone changes may in particular be involved in disease development later in life [5]. In addition, there was a large variation in the tissue chosen to be investigated. As highlighted by Geraghty et al. [18], the epigenetic signatures may vary according to the specific tissue type under investigation, which can make it difficult to interpret and compare the response to the different exposures.

Several of the included studies in the present review may also be prone to various flaws from a methodological point of view. While all studies considered potential confounding factors either by design or by multivariate analyses, the covariates considered across the included studies were dissimilar. Similarly, several of the studies did not adjust for potential important confounding variables such as dietary factors (e.g., folic acid) known to alter epigenetic markers and other environmental factors (e.g., physical activity). A point worth mentioning here is the correlation between mothers and their offspring in terms of genetics. DNA methylation has been shown to be strongly influenced by genetic variation [38], which can also result in bias if not adjusted for—especially when it comes to an observational design, which constitutes the majority of the studies in this review. Further, cord blood and tissue include several types of cells which may encompass variations that are important to adjust for. This was only carried out in the study by Antoun et al. [17] (See Table 1).

In addition to the lack of adjustment for potential important confounding factors, several of the included cohort studies did not provide sufficient information on the completeness of the follow-up period. Thus, whether these studies are prone to selection bias is difficult to determine. Similarly, most of the included cohort studies used self-report exposure measures (e.g., Food Frequency Questionnaire) or exposure data that were dependent on the memory of the participants (e.g., 24 h dietary recall), which may have resulted in information bias (see Table 3).

It is also important to note that in most of the studies included in this review, power calculations have not been performed based on the epigenetic outcome, but on the primary outcomes in the studies, examining the effect of diet or exercise on glycemic control, GWG, GDM development, LGA infants, etc. Therefore, the sample sizes in the studies differ substantially and caution should be taken in drawing causative conclusions.

We identified no studies examining the paternal influence on the epigenetics in our review. A systematic review focusing on epigenetic effects (DNA methylation) of paternal and maternal obesity found three studies examining the paternal influence [2]. All three studies identified possible paternal influence on epigenetics. Therefore, the father’s pre-conceptional lifestyle, including diet and level of physical activity, may be a confounder not considered in any of the studies included in this review.

As mentioned above, the first trimester is a crucial period during pregnancy for epigenomic programming and development of the fetus. Therefore, the fetus is especially vulnerable to environmental factors in the first trimester [16]. Not all studies included in the current review investigated the exposure during the first trimester since recruitment often took place during the first or second trimester. Accordingly, most of the studies did not examine the exposures during the most influential phase of pregnancy, and in the RCT studies, participants were typically recruited in the second trimester.

Despite the heterogenic nature of the studies and the varying sample sizes, most of the studies showed significant differences in DNA methylation levels between groups, which may indicate that maternal lifestyle factors during pregnancy associate with epigenetic changes in the offspring. The variety in studies in relation to design, exposure, examined tissue and outcome measurements can to some extent therefore also be considered as a strength, as the findings were consistent in most of the studies. Due to the small number of studies available, we were not able to further narrow the literature search. More uniform studies in terms of particular exposures, tissues and outcomes would however make it easier to compare studies.

Future studies designed to examine lifestyle, diet or physical activity to reduce the risk of later lifestyle disease in offspring should focus on interventions at an early stage in fetal life [4]. Interventions early in pregnancy and even before pregnancy and peri-conceptionally could be the way to reduce the risk of obesity and disease later in life, through epigenetic changes.

## 5. Conclusions

The present review found that healthy lifestyle changes, i.e., exercise and diet, during pregnancy could have an impact on DNA methylation and miRNA in placenta and offspring, but the research field is evolving, and solid evidence-based advice is still lacking. There are indications that a healthy lifestyle during pregnancy is important in order to reduce the risk of childhood obesity, diabetes and inflammatory conditions through epigenetic actions. However, as the available studies are both few and heterogenic, there is an urgent need for larger, well-performed studies to elucidate the specific epigenetic fetal markers that are affected by maternal and perhaps paternal lifestyle before and shortly after conception, in order to point out measures to prevent non-communicable diseases such as childhood obesity and chronic metabolic diseases in the future generation.

## Figures and Tables

**Table 1:** Study characteristics.

Gene-Specific Approach							
Study	Design	Participants	Location	Confounding Variables	Tissue	Tool	Comparison
Daniels et al., 2020 [12]	Cohort	135 Mother–offspring pairs. Mainly white mothers (72.5%)	USA	Maternal age, rs2167270 genotype, pre-pregnancy obesity and gestational diabetes	Placenta	Pyrosequencing, Pyromark MD (Qiagen)	Association between maternal intake of carbohydrates and added sugar intake and DNA methylation
Drake et al., 2012 [13]	Cohort	34 Mother–offspring pairs (40 years old)	Scotland	Neonatal anthropometry: Gestational age at delivery, parity, sex and maternal antenatal BMI Adult anthropometry: blood pressure	Peripheral blood	Pyrosequencing	Association of maternal adherence to dietary advice of increased protein and reduced carbohydrate intake during pregnancy and DNA methylation
Godfrey et al., 2011 [14]	2 × Cohort ^1^	78/239 Mother–offspring pairs. In the explorative cohort (PAH), the included mothers were primarily white	UK	Sex, maternal age, adiposity and smoking during pregnancy	Cord blood	Sequenom MassARRAY	Association between maternal intake of carbohydrate, fat and protein and DNA methylation
Gonzalez-Nahm et al., 2017 [15]	Cohort	390 Mother–offspring pairs	USA	Maternal pre-pregnancy BMI, maternal age, maternal smoking during pregnancy and maternal education	Cord blood	Pyrosequencing	Association between maternal adherence to a Mediterranean diet and DNA methylation. Participants were grouped to either low adherence, medium or high adherence
Marshall et al., 2018 [4]	Cohort	42 Mother–offspring pairs	USA	NA ^2^	Infant blood spots	Pyrosequencing	Association between maternal physical activity and DNA methylation. Participants were grouped according to leisure-time physical activity. ‘High active’ group: average 637.5 min per week versus ‘low active’ group: average 59.5 min per week
McCullough et al., 2015 [6]	Cohort	484 Mother–offspring pairs. 7% mothers with (gestational diabetes) GDM. 46% Black, 69% White, 29% Hispanic	USA	Race/ethnicity and preterm birth	Cord blood	Pyrosequencing (Pyromark Q96 MD pyrosequencer (Qigen))	Association between maternal sedentary time and DNA methylation. Participants were grouped according to physical activity level (non-sedentary time) in quartiles
McCullough et al., 2017 [3]	Cohort	1057 Mother–offspring pairs. 43% Black, 34% White and 23% Hispanic	USA	Maternal race/ethnicity, BMI at last menstrual period and maternal smoking	Cord blood	Pyrosequencing	Association between maternal intake of a pro-inflammatory diet and DNA methylation. Participants were grouped in dietary inflammatory index (DII) quartiles
Miyaso et al., 2017 [16]	Cohort	91 Mother–offspring pairs. Majority Asian	Japan	Maternal BMI, maternal socioeconomic status (education and income), newborn sex and head circumference	Umbilical cord tissue	MS-HRM analysis	Association between maternal calorie and carbohydrate intake and DNA methylation
Genome-wide approach							
**Study**	**Design**	**Participants**	**Location**	**Confounding Variables**	**Tissue**	**Tool**	**Comparison**
Antoun et al., 2020 [17]	RCT	294 Mother–offspring pairs. Pre-pregnancy BMI ≥ 30 kg/m^2^–72.3% White, 5.2% Asian, 17.7% Black, 4.8% others	UK	Maternal age, predicted values for white blood cells and nucleated red blood cells composition, smoking, ethnicity, parity and neonate sex	Cord blood	Illumina Human Methylation EPIC Beadchip (850 K)	Effect of a low glycemic index diet, reduced saturated fat intake and increased physical activity intervention during pregnancy compared with standard antenatal care on DNA methylation
Geraghty et al., 2020 [18]	RCT	60 Mother–offspring pairs (5 years old). Inclusion criteria: mothers who had previously given birth to a macrosomic infant. 96.8% White mothers	Ireland	Offspring sex	Saliva	Illumina Human Methylation EPIC Beadchip (850 K)	Effect of a low glycemic index diet intervention during pregnancy (received dietary education session with a research dietitian with dietary advice regarding healthy eating in general and specifically about a low glycemic index diet) compared to antenatal care and no specific dietary advice on DNA methylation
Geraghty et al., 2018 [19]	RCT	60 Mother–offspring pairs Included mothers who had previously given birth to a macrosomic infant. Mean BMI in early pregnancy: 25.75. 96.8% white	Ireland	Offspring sex and gestational age	Cord blood serum	Illumina Human Methylation EPIC Beadchip (850 K)	Effect of a low glycemic index diet intervention during pregnancy (received dietary education session with a research dietitian with dietary advice regarding healthy eating in general and specifically about a low glycemic index diet) compared to antenatal care and no specific dietary advice on DNA methylation
Gomez Ribot et al., 2020 [20]		45 Mother–offspring pairs. 30 Mothers had GDM ^3^	Argentina	NA ^4^	Placenta	TaqMan detection system	Effect of an intervention with olive oil on miRNA in women with GDM (15 received three tablespoons of olive oil and 15 received only none to one tablespoon a day) and compared to healthy controls (*n* = 15). All were advised on healthy eating
Jönsson et al., 2021 [21]	RCT	208 Mothers with obesity and their offspring. Mean pre-pregnancy BMI (SD): Intervention: 34.19 (4.00) kg/m^2^; control: 34.36 (3.98) kg/m^2^	Denmark	Maternal age, pre-pregnancy BMI, GWG, GA and offspring sex	Cord blood	Illumina Infinium HumanMethylation450 BeadChips	Effect of a physical activity assessed with pedometer and dietary advice (PA + D), or only physical activity (PA) compared to a control group receiving standard of care on DNA methylation
Thakali et al., 2020 [22]	Cohort	150 Mother–offspring pairs; 72 mothers with normal weight and 78 mothers with overweight/obesity	USA	Maternal age at delivery, infant sex and delivery mode	Placenta	Reduced representation bisulfite sequencing (RRBS)	Association between maternal intake of carbohydrate, protein and fat and DNA methylation
Trumpff et al., 2021 [23]	Cohort	73 Mother–offspring pairs. 2% of mothers had diabetes mellitus. 68% Hispanic, 32% Not Hispanic	USA	Birthweight, sex, pregnancy complications, maternal age, average maternal calorie intake and maternal pre-pregnancy weight	Placenta	Illumina Human Methylation 450 K Beadchip	Association between maternal intake of sugar (in teaspoons equivalents) and DNA methylation
Yan et al., 2019 [24]	Cohort	24/153 ^5^ Mothers with overweight 33.3% had GDM in case group, 16.7% had GDM in control group	China	Maternal age, BMI, Homeostatic Model Assessment for Insulin Resistance (HOMA-IR), dietary GI at baseline, GDM, gestational age at delivery and gestational weight gain	Placenta	Illumina Human Methylation 450 K Beadchip	Association between changes in maternal glycemic index intake and DNA methylation

^1^ One exploratory (PAH) and one replication cohort (SWS). ^2^ No covariates were included in the analyses due to the matched design and small sample size. ^3^ 15 mothers with GDM in intervention, 15 mothers with GDM in control group (30 randomized) and 15 healthy controls. ^4^ No adjustments for potential confounding variables were performed. BMI, body mass index; DII, dietary inflammatory index; GA, gestational age; GDM, gestational diabetes mellitus; GWG, gestational weight gain; RCT, Randomized Controlled Trials; UK, United Kingdom; USA, United States of America. ^5^ Discovery sample/replication sample.

**Table 2:** Outcome measures and main findings according to exposure.

Study	Outcome Measures	Main Findings
Physical activity		
Antoun et al., 2020 [17]	Genome-wide DNA methylation changes in cord blood of offspring born to mothers with GDM who participated in a RCT of impact of low glycemic diet, reduced saturated fat and increased physical activity intervention during pregnancy.	No overall association between intervention and differential methylation in the cord blood. However, a total of 87% of the GDM and 77% of the 1 h glucose-associated differentially methylated CpGs had smaller effect sizes in the intervention group compared to the standard care arm.
Jönsson et al., 2021 [21]	Genome-wide DNA methylation in cord blood of offspring born to mothers participating in a RCT of impact of physical activity ± dietary intervention (low-fat and low-energy Mediterranean-style diet) in pregnancy. Also, association between intervention, methylation changes and offspring body composition.	DNA methylation was altered at 379 sites annotated to 370 genes in the intervention group versus control group (false discovery rate (FDR) < 0.05). These 370 genes are overrepresented in gene ontology terms, including response to fatty acids and adipose tissue development. Methylation of 17 sites was found to partially mediate the effect of lifestyle intervention on lean body mass in the offspring (FDR < 0.05).
Marshall et al., 2018 [4]	Association between maternal physical activity level during pregnancy and global DNA methylation as well as methylation at candidate gene level in blood from newborn. The mothers were divided into ‘high active’ (HA) and a ‘low active’ (LA).	No effect of physical activity on global DNA methylation. There were no differences between HA and LA mothers for LINE-1. However, the P2 promoter within the IGF2 gene was significantly higher in the LA group compared with HA (*p* = 0.045)
McCullough et al., 2015 [6]	Association between maternal non-sedentary time during pregnancy and DNA methylation at four DMRs in cord blood from offspring.	Non-sedentary time decreased methylation at the PLAG1 DMR and was found to, in part, account for an association between maternal physical activity and offspring birthweight.
Carbohydrate intake		
Daniels et al., 2020 [12]	Association between DNA methylation changes at CPGs localized in the promotor region of the placenta leptin gene and maternal intake of calorie, carbohydrates, fat, protein and added sugar during pregnancy.	Lower levels of leptin methylation were associated with greater intake of carbohydrates after controlling for leptin SNP genotype (*p* < 0.05). Total caloric intake was also associated with placenta leptin methylation (*p* < 0.05), however after controlling for relevant covariates, significance diminished to trend-level. There were no associations of methylation and intake of protein (*p* > 0.05) or fat (*p* > 0.05).
Godfrey et al., 2011 [14]	Association between DNA methylation at CPGs of selected candidate genes in cord blood and maternal carbohydrate, fat and protein intake during pregnancy.	Higher methylation of RXRA chr9:136355885 was associated with lower maternal carbohydrate intake. Fat and protein intake were not associated with DNA methylation changes. Maternal intake of fat and protein were not associated with DNA methylation level.
Miyaso et al., 2017 [16]	Any association between methylation level at the H19 DMR in umbilical cord tissue and maternal diet.	Calorie intake of less than 1000 kcal/day was related to lower methylation levels at the H19 DMR (*p* = 0.013). The study did not find any significant effect of carbohydrate intake (OR = 1.28, *p* = 0.067).
Thakali et al., 2020 [22]	Association between genome-wide DNA methylation changes in placenta and maternal fat, carbohydrate, protein and saturated fat intake during pregnancy.	Maternal saturated fat intake was significantly associated with placental methylation at 302 of approximately 300,000 CpGs. Maternal carbohydrate, protein and total fat intake were significantly associated with methylation of 12, 14 and 28 CpGs, respectively. However, no maternal diet predictor variable was significantly associated with placental DNA methylation after adjusting for multiple testing.
Glycemic index		
Antoun et al., 2020 [17]	See above	See above
Geraghty et al., 2018 [19]	Genome-wide DNA methylation changes in cord blood of offspring born to mothers who participated in a RCT with a low glycemic diet intervention in pregnancy.	Low glycemic intervention during pregnancy induced widespread DNA methylation changes. However, none strong enough to pass multiple testing.
Geraghty et al., 2020 [18]	Genome-wide DNA methylation changes at age 5 in offspring born to mothers who participated in a RCT with a low glycemic diet intervention in pregnancy. DNA was derived from saliva from both offspring exposed to the intervention and unexposed controls. In addition, DNA methylation and body composition at age 5 were studied.	No association was found between offspring DNA methylation and the dietary intervention in pregnancy (adjusted *p* < 0.05). However, applying an un-adjusted *p* < 0.05 identified 22,181 CPGs. The Top 1000 highest-ranking CPGs were selected for gene pathway analysis (most of them, corresponding to 60%, were hypermethylated). The gene pathway analysis showed enrichment with regards to insulin functioning.
Yan et al., 2019 [24]	Association between genome-wide DNA methylation changes in the placenta and maternal glycemic index changes during pregnancy.	Methylation levels of cg17586860 and cg18197392 in the 5ÚTR region of SSTR4 were negatively correlated with changes in carbohydrate intake and glycemic load across gestation.
Sugar intake		
Daniels et al., 2020 [12]	See above	See above
Trumpff et al., 2021 [23]	Association between genome-wide DNA methylation changes in the placenta and maternal added sugar intake during pregnancy.	No individual CpGs achieved significance for altered methylation as a function of added sugar intake at each trimester or across pregnancy. A secondary analysis revealed that added sugar intake in the third trimester was associated with increased DNA methylation of a cluster of 8 CpGs within the ADD2 gene.
Mediterranean diet		
Gonzalez-Nahm et al. [25]	Association between DNA methylation changes in infant cord blood leukocytes at the following DMRs: MEG3-IG, MEG3, IGF2, H19, PLAGL1, MEST, NNAT, PEG3 and SGCE/PEG10, and maternal adherence to a Mediterranean diet.	Female infants of mothers with a low adherence to a Mediterranean diet had higher odds of hypo-methylation at the MEG3-IG DMR.
Jönsson et al. [21]	See above	Se above
Fat intake		
Antoun et al., 2020 [17]	See above	See above
Daniels et al., 2020 [12]	See above	See above
Godfrey et al., 2011 [14]	See above	See above
Thakali et al., 2020 [22]	See above	See above
Other exposures		
Drake et al. [13]	Association between DNA methylation at CPGs localized in the GR, HSD2 and IGF2 in blood from adult offspring (40 years of age) and maternal intake of ‘an unbalanced diet’ consisting of a high meat intake and a low carbohydrate intake during pregnancy.	Offspring whose mothers reported a high meat/fish and vegetable intake combined with a low bread/potato intake had higher mean methylation at GR exon 1F. Methylation was also increased at a specific CpG site in H2D2 region 2 if the mothers had an increased meat (*p* = 0.03) and fish intake (*p* = 0.04).
Gomez Ribot et al. [20]	The relative expression of microRNA, miR-130a and miR-518d, in placenta of offspring born to mothers with GDM who participated in a RCT of the impact of three tablespoons of olive oil as a supplement to their regular diet during pregnancy.	No changes were found in the expression of miR-130a, but 518d expression was found to be increased in the placenta of GDM mothers but reduced in those GDM mothers receiving the intervention (*p* = 0.009).
McCullough et al., 2017 [3]	Association between methylation changes at the following DMRs: IGF2, H19, MEG3, PEG3, MEST, SGCE/PEG10, NNAT and PLAGL1 in cord blood in offspring and maternal intake of a pro-inflammatory diet during pregnancy.	No association between maternal intake of a pro-inflammatory diet and methylation of the DMRs was found.

**Table 3:** Quality Assessment of Cohort Studies.

Study	Did the Study Address a Clearly Focused Issue?	Was the Cohort Recruited in an Acceptable Way?	Was the Exposure Accurately Measured to Minimize Bias?	Was the Outcome Accurately Measured to Minimize Bias?	Have the Authors Identified All Important Confounding Factors?	Have They Accounted for the Confounding Factors in the Design and/or Analysis?	Was the Follow-up of Subjects Complete Enough?	Was the Follow-up of Subjects Long Enough?	Are the Results Precise?	Do You Believe the Results?	Can the Results Be Applied to the Local Population?	Do the Results of This Study Fit with Other Available Evidence?	Are there Implications of This Study for Practice?
Daniels et al., 2020 [12]	Yes	Can’t tell	Yes	Yes	Yes	Yes	Can’t tell	Can’t tell	Yes	Yes	Can’t tell	Yes	Can’t tell
Drake et al., 2021 [13]	Yes	Yes	No	Yes	Yes	Yes	Yes	Yes	Yes	Yes	Yes	Yes	Yes
Godfrey et al. [14]	Yes	Yes	No	Yes	Yes	Yes	Yes	Yes	Yes	Yes	Yes	Yes	Yes
Gonzalez-Nahm et al., 2017 [25]	Yes	Yes	No	Yes	Yes	Yes	Yes	Yes	Yes	Yes	Yes	Yes	Yes
Marshall et al., 2018 [4]	Yes	Yes	Yes	Yes	Can’t tell	Yes	Can’t tell	Yes	Can’t tell	No	No	No	No
McCullough et al., 2015 [6]	Yes	Yes	Yes	Yes	No	Yes	Can’t tell	Yes	Yes	Yes	Yes	Yes	Yes
McCullough et al., 2017 [3]	Yes	Yes	Yes	Yes	No	Yes	Can’t tell	Yes	Yes	Yes	Yes	Yes	Yes
Miyaso et al., 2017 [16]	Yes	Can’t tell	Can’t tell	Yes	No	Yes	Can’t tell	Yes	No	Yes	Yes	Yes	Yes
Thakali et al., 2020 [22]	Yes	Yes	Yes	Yes	No	Yes	Can’t tell	Yes	Yes	Yes	Yes	Yes	Yes
Trumpff et al., 2021 [23]	Yes	Yes	No	Yes	Yes	Yes	Yes	Yes	Yes	Yes	Yes	Yes	Yes
Yan et al., 2019 [24]	Yes	Yes	No	Yes	No	Yes	Yes	Yes	Yes	Yes	Yes	Yes	Yes

The study quality and bias assessment was assessed using the Critical Appraisal Skills Program (CASP) Cohort Study Checklist [11].

**Table 4:** Quality Assessment of Randomized Controlled Trails.

Study	Risk of Bias Arising from Randomization Process	Risk of Bias Due to Deviations from Intended Interventions	Risk of Bias due to Missing Outcome Data	Risk of Bias in the Measurement of the Outcome	Risk of Bias in the Selection the Reported Result	Overall Risk of Bias
Geraghty et al., 2020 [18]	Low	Some concerns	Low	Low	Some concerns	Some concerns
Geraghty et al., 2018 [19]	Low	Some concerns	Low	Low	Some concerns	Some concerns
Gomez Ribot et al., 2020 [20]	Some concerns	Low	Low	Low	Low	Low
Jönsson et al., 2021 [21]	Some concerns	Some concerns	Low	Low	Low	Low
Antoun et al., 2020 [17]	Low	Low	Low	Low	Some concerns	Low

The study quality and bias assessment was assessed using the revised Cochrane risk-of-bias tool for randomized trials (RoB2) [10].

## Abbreviations

ADD2	Adducin 2
BMI	Body mass index
DII	Dietary inflammatory index
DMR	Differentially methylated region
CASP	Critical Appraisal Skills Program
CpG	Cytosine-phosphate-guanine
FDR	False discovery rate
GA	Gestational age
GDM	Gestational diabetes mellitus
GWG	Gestational weight gain
GR	Glucocorticoid receptor
HA	High active
HSD2	11β-hydroxysteroid dehydrogenase type 2
IGF-2	Insulin-like growth factor 2
LA	Low active
LGA	Large for gestational age
LINE	Long interspersed nuclear elements
LTPA	Leisure-time physical activity
MEG3	Maternally expressed 3
MEST	Mesoderm-specific transcript
miRNA	MicroRNA
NNAT	Neuronatin
OGTT	Oral glucose tolerance test
PEG	Polyethylen glycol
PLAG1	Pleomorphic adenoma gene 1
RCT	Randomized Controlled Trial
RoB	Revised Cochrane risk-of-bias tool
RXRs	Retinoid X receptors
SGCE	Sarcoglycan epsilon
SNP	Single-nucleotide polymorphism
SST	Somastostetin
SSTR4	Somatostatin receptor 4
TNDM	Transient neonatal diabetes mellitus
UC	Umbilical cord
UK	United Kingdom
USA	United States of America
5′ UTR	5′ Untranslated region

## Appendix A. Literature Search

**PubMed on 10 March 2021.**

((((((((((((((((“Pregnancy”[Mesh]) OR (“Mothers”[Mesh])) OR (“Infant”[Mesh])) OR (“Paternity”[Mesh])) OR (pregnancy[Text Word])) OR (neonatal[Text Word])) OR (infant[Text Word])) OR (maternal*[Text Word])) OR (maternit*[Text Word])) OR (paternit*[Text Word])) OR (paternal*[Text Word])) OR (preconception[Text Word])) OR (periconception[Text Word])) OR (offspring[Text Word])) AND (((((((“Epigenome”[Mesh]) OR (“Epigenomics”[Mesh])) OR (“MicroRNAs”[Mesh])) OR (“DNA Methylation”[Mesh])) OR (epigenom*[Text Word])) OR (microRNA*[Text Word])) OR (DNA methylation[Text Word]))) AND ((((((((((“Food”[Mesh]) OR (“Diet”[Mesh])) OR (“Nutrients”[Mesh])) OR (“Exercise”[Mesh])) OR (food[Text Word])) OR (nutrient*[Text Word])) OR (nutrition*[Text Word])) OR (diet*[Text Word])) OR (exercise[Text Word])) OR (physical activity[Text Word]))) NOT ((“Animals”[Mesh]) NOT (“Humans”[Mesh]))

1107 Results.

**Embase on 10 March 2021.**

(‘pregnancy’/exp OR ‘infant’/exp OR ‘mother’/exp OR ‘paternity’/exp OR ‘preconception’/exp OR ‘periconception’/exp OR pregnancy:ab,ti,kw OR preconception:ab,ti,kw OR periconception:ab,ti,kw OR maternal*:ab,ti,kw OR maternit*:ab,ti,kw OR paternit*:ab,ti,kw OR paternal*:ab,ti,kw OR offspring*:ab,ti,kw OR infant*:ab,ti,kw OR neonatal*:ab,ti,kw) AND (‘epigenome’/exp OR ‘epigenetics’/exp OR ‘microrna’/exp OR ‘dna methylation’/exp OR epigenom*:ab,ti,kw OR epigenetic*:ab,ti,kw OR microrna*:ab,ti,kw OR ‘dna methylation’:ab,ti,kw) AND (‘food’/exp OR ‘diet’/exp OR ‘nutrient’/exp OR ‘exercise’/exp OR food:ab,ti,kw OR nutrient*:ab,ti,kw OR nutrition*:ab,ti,kw OR diet*:ab,ti,kw OR exercise:ab,ti,kw OR ‘physical activity’:ab,ti,kw) NOT ([animals]/lim NOT [humans]/lim) AND (‘article’/it OR ‘article in press’/it OR ‘erratum’/it OR ‘review’/it)

1961 Results.

**Cochrane: on 10 March 2021.**

#1 (pregnancy OR neonatal OR infant OR maternal OR maternit* OR paternit* OR paternal OR preconception OR periconception OR offspring):ti,ab,kw (Word variations have been searched):  120,264
#2 MeSH descriptor: [Pregnancy] explode all trees:   22,290
#3 MeSH descriptor: [Mothers] explode all trees: 1853
#4 MeSH descriptor: [Infant] explode all trees:  32,413
#5 MeSH descriptor: [Paternalism] explode all trees:  8
#6 #1 OR #2 OR #3 OR #4 OR #5:  120,760
#7 (epigenom* OR microRNA* OR DNA methylation):ti,ab,kw:  1952
#8 MeSH descriptor: [Epigenomics] explode all trees:  8
#9 MeSH descriptor: [Epigenomics] explode all trees:  8
#10 MeSH descriptor: [MicroRNAs] explode all trees:  173
#11 MeSH descriptor: [DNA Methylation] explode all trees:  236
#12 #7 OR #8 OR #9 OR #10 OR #11:  1952
#13 (food OR nutrient* OR nutrition* OR diet* OR exercise OR physical activity):ti,ab,kw:  234,523
#14 MeSH descriptor: [Food] explode all trees:  34,794
#15 MeSH descriptor: [Diet] explode all trees:  18,618
#16 MeSH descriptor: [Nutrients] explode all trees:  5254
#17 MeSH descriptor: [Exercise] explode all trees: 25,067
#18 #13 OR #14 OR #15 OR #16 OR #17:   244,447
#19 #6 AND #12 AND #18: 94
#20 MeSH descriptor: [Animals] explode all trees: 599,446
#21 MeSH descriptor: [Humans] explode all trees: 599,386
#22 #20 NOT #21:  60
#23 #19 NOT #22:  94

94 Results

**In total: 3162 Results**

**After removal of duplicates: 2467 Results.**

## Appendix B. Example of a Search Block (PubMed)

**Table A1:** Search strategy in PubMed.

**BLOCK 1**	**AND**	**BLOCK 2**	**AND**	**BLOCK 3**	**NOT**	**BLOCK 4**
Pregnancy		Food		Epigenome		Animals
Mothers		Diet		Epigenomics		
Infant		Nutrients		MicroRNAs		
Paternity		Exercise		DNA methylation		
neonatal		Nutrient		Epigenom*		
maternal		Nutrition		microRNA*		
maternit*		diet				
paternit*		Physical activity				
paternal*						
preconception						
Periconception						
offspring						

The search words within the columns were combined with “OR”, and across the columns the words were combined with “AND” and “NOT”.

## Appendix C. Template to Extract Relevant Study Data

**Study/Data**	**Antoun et al., 2020**	**Daniels et al., 2020**	**….**
**Basic**			
Publication year			
Country/Location			
Study design			
**Participants**			
Number of participants (*n*)			
Sociodemographic data			
Health status/diseases			
Ethnic groups			
Age of offspring/child			
Gender of infant			
**Intervention/Exposures**			
Dietary pattern			
Dietary intake			
Physical activity level			
Control group/comparison			
**Outcome methodology/tool**			
The methodology used to assess DNA methylation			
The methodology used to assess changes in Noncoding RNA, including microRNA			
Tissue			
**Confounding variables**			
**Results**			
DNA methylation levels			
Reported global/gene-specific methylation			
Changes in DNA methylation between groups (hypo- or hyper-methylation)			
Log fold changes used in comparison between groups			
Changes in microRNA			
Main results			
**Statistical analyses employed in the study**			
**Conclusion**			

## References

[1] Marciniak A., Patro-Małysza J., Kimber-Trojnar Z., Marciniak B., Oleszczuk J., Leszczyńska-Gorzelak B. (2017). Fetal programming of the metabolic syndrome. Taiwan. J. Obstet. Gynecol..

[2] Dunford A.R., Sangster J.M. (2017). Maternal and paternal periconceptional nutrition as an indicator of offspring metabolic syndrome risk in later life through epigenetic imprinting: A systematic review. Diabetes Metab. Syndr. Clin. Res. Rev..

[3] McCullough L.E., Miller E.E., Calderwood L.E., Shivappa N., Steck S.E., Forman M.R., Mendez M.A., Maguire R., Fuemmeler B.F., Kollins S.H. (2017). Maternal inflammatory diet and adverse pregnancy outcomes: Circulating cytokines and ge-nomic imprinting as potential regulators?. Epigenetics.

[4] Marshall M.R., Paneth N., Gerlach J.A., Mudd L.M., Biery L., Ferguson D., Pivarnik J.M. (2018). Differential methylation of insulin-like growth factor 2 in offspring of physically active pregnant women. J. Dev. Orig. Health Dis..

[5] Hjort L., Novakovic B., Grunnet L.G., Maple-Brown L., Damm P., Desoye G., Saffery G. (2019). Diabetes in pregnancy and epigenetic mechanisms-how the first 9 months from conception might affect the child’s epigenome and later risk of disease. Lancet Diab. Endocrinol..

[6] McCullough L.E., Mendez H.A., Miller E.E., Murtha P.A., Murphy S.K., Hoyo C. (2015). Associations between prenatal physical activity, birth weight, and DNA methylation at genomi-cally imprinted domains in a multiethnic newborn cohort. Epigenetics.

[7] Opsahl J.O., Moen G.-H., Qvigstad E., Bottcher Y., Birkeland K.I., Sommer C. (2021). Epigenetic signatures associated with maternal body mass index or gestational weight gain: A system-atic review. J. Dev. Orig. Health Dis..

[8] Page M.J., McKenzie J.E., Bossuyt P.M., Boutron I., Hoffmann T.C., Mulrow C.D., Shamseer L., Tetzlaff J.M., Akl E.A., Brennan S.E. (2021). The PRISMA 2020 statement: An updated guideline for reporting systematic reviews. BMJ.

[9] Covidence (2019). Covidence—Better Systematic Review Management. https://www.covidence.org/.

[10] Cochrane Collaboration RoB 2: A Revised Cochrane Risk-Of-Bias Tool for Randomized Trials. https://methods.cochrane.org/bias/resources/rob-2-revised-cochrane-risk-bias-tool-randomized-trials.

[11] CRITICAL APPRAISAL SKILLS PROGRAMME (CASP) CASP Checklists 2021. https://casp-uk.net/casp-tools-checklists/.

[12] Daniels T.E., Sadovnikoff A.I., Ridout K.K., Lesseur C., Marsit C.J., Tyrka A.R. (2020). Associations of maternal diet and placenta leptin methylation. Mol. Cell. Endocrinol..

[13] Drake A.J., McPherson R.C., Godfrey K.M., Cooper C., Lillycrop K.A., Hnason M.A., Meehan R.R., Seckl J.R., Reynolds R.M. (2012). An unbalanced maternal diet in pregnancy associates with offspring epigenetic changes in genes con-trolling glucocorticoid action and foetal growth. Clin. Endocrinol..

[14] Godfrey K.M., Sheppard A., Gluckman P.D., Lollycrop K.A., Burdge G.A., McLean C., Rodfoard E., Slater-Jefferies S., Garratt E., Crozier S.R. (2011). Epigenetic gene promoter methylation at birth is associated with child’s later adiposity. Diabetes.

[15] González C.R., González B. (2020). Exploring the Stress Impact in the Paternal Germ Cells Epigenome: Can Catecholamines Induce Epigenetic Reprogramming?. Front. Endocrinol..

[16] Miyaso H., Sakurai K., Takase S., Eguchi A., Watanabe M., Fukuoka H., Mori C. (2017). The methylation levels of the H19 differentially methylated region in human umbilical cords reflect newborn parameters and changes by maternal environmental factors during early pregnancy. Environ. Res..

[17] Antoun E., Kitaba N.T., Titcombe P., Dalrymple K.V., Garratt E.S., Barton S.J., Murray R., Seed P.T., Holbrook J.D., Kobor M.S. (2020). Maternal dysglycaemia, changes in the infant’s epigenome modified with a diet and physical activity intervention in pregnancy: Secondary analysis of a randomised control trial. PLoS Med..

[18] Geraghty A.A., Sexton-Oates A., O’Brien E.C., Saffery R., McAuliffe F.M. (2020). Epigenetic Patterns in Five-Year-Old Children Exposed to a Low Glycemic Index Dietary Intervention during Pregnancy: Results from the ROLO Kids Study. Nutrition.

[19] Geraghty A.A., Saxton-Oates A., O’Brien E.C., Alberdi G., Frasquet P., Saffery R., McAuliffe F.F. (2018). A low glycaemic index diet in pregnancy induces DNA methylation variation in blood of new-borns: Results from the ROLO randomised controlled trial. Nutrients.

[20] Gomez Ribot D., Diaz G., Fazion M.V., Gomez H.L., Fornes D., Macchi S.B., Gresta C.A., Capobianco E., Jawerbaun A. (2020). An extra virgin olive oil-enriched diet improves maternal, placental, and cord blood parameters in GDM pregnancies. Diabetes Metabol. Res. Rev..

[21] Jönsson J., Renault K.M., García-Calzón S., Perfilyev A., Estampador A.C., Nørgaard K., Lind M.V., Vaag A., Hjort L., Michaelsen K.F. (2021). Lifestyle Intervention in Pregnant Women with Obesity Impacts Cord Blood DNA Methylation, Which Associates with Body Composition in the Offspring. Diabetes.

[22] Thakali K.M., Zhong Y., Cleves M., Andres A., Shankar K. (2020). Associations between maternal body mass index and diet composition with placental DNA methyla-tion at term. Placenta.

[23] Trumpff C., Sturm G., Picard M., Foss S., Lee S., Feng T., Cardenas A., McCormack C., Champagne F.A., Monk C. (2021). Added sugar intake during pregnancy: Fetal behavior, birth outcomes, and placental DNA methylation. Dev. Psychobiol..

[24] Yan W., Zhang Y., Wang L., Yang W., Li C., Gu P., Xia Y., Yan J., Shen Y., Zhao Q. (2019). Maternal dietary glycaemic change during gestation influences insulin-related gene methylation in the placental tissue: A genome-wide methylation analysis. Genes Nutr..

[25] Gonzalez-Nahm S., Mendez M., Robinson W., Murphy S.K., Hoyo C., Hogan V., Rowley D. (2017). Low maternal adherence to a Mediterranean diet is associated with increase in methylation at the MEG3-IG differentially methylated region in female infants. Environ. Epigenetics.

[26] Antoun E., Kitaba P., Titcombe P., Dalrymple K., Seed P.T., White S.L., Burdge G.C., Poston L., Godfrey K.M., Lillycrop K.A. (2019). Maternal gestational diabetes is associated with changes in the infant methylome. Diabet. Med..

[27] Godfrey K.M., Inskip H.M., Hanson M.A. (2011). The Long-Term Effects of Prenatal Development on Growth and Metabolism. Semin. Reprod. Med..

[28] Mackay D.J., Temple I.K. (2010). Transient neonatal diabetes mellitus type 1. Am. J. Med. Genet. Part C Semin. Med. Genet..

[29] Bao D., Yuan R.X., Zhang Y. (2020). Effects of lncRNA MEG3 on proliferation and apoptosis of gallbladder cancer cells through regulating NF-κB signaling pathway. Eur. Rev. Med. Pharmacol. Sci..

[30] Chang W.-W., Zhang L., Yao X.-M., Chen Y., Zhu L.-J., Fang Z.-M., Zhao Y., Yao Y.-S., Jin Y.-L. (2020). Upregulation of long non-coding RNA MEG3 in type 2 diabetes mellitus complicated with vascular disease: A case–control study. Mol. Cell. Biochem..

[31] Buccarelli M., Lulli V., Giuliani A., Signore M., Martini M., D’Alessandris Q.G., Giannetti S., Novelli A., Ilari R., Giurato G. (2020). Deregulated expression of the imprinted DLK1-DIO3 region in glioblastoma stemlike cells: Tumor suppressor role of lncRNA MEG3. Neuro Oncol..

[32] Alipoor B., Parvar S.N., Sabati Z., Ghaedi H., Ghasemi H. (2020). An updated review of the H19 lncRNA in human cancer: Molecular mechanism and diagnostic and therapeutic importance. Mol. Biol. Rep..

[33] Yaghootkar H., Zhang Y., Spracklen C.N., Karaderi T., Huang L.O., Bradfield J., Schurmann C., Fine R.S., Preuss M.H., Kutalik Z. (2020). Genetic Studies of Leptin Concentrations Implicate Leptin in the Regulation of Early Adiposity. Diabetes.

[34] Li J., Gao Y., Yu T., Lange J.K., LeBoff M.S., Gorska A., Luu S., Zhou S., Glowacki J. (2019). Obesity and leptin influence vitamin D metabolism and action in human marrow stromal cells. J. Steroid Biochem. Mol. Biol..

[35] Chen L., Wu L., Zhu L., Zhao Y. (2018). Overview of the structure-based non-genomic effects of the nuclear receptor RXRα. Cell. Mol. Biol. Lett..

[36] Chen Y., Ma G., Hu Y., Yang Q., Deavila J.M., Zhu M.-J., Du M. (2021). Effects of Maternal Exercise During Pregnancy on Perinatal Growth and Childhood Obesity Outcomes: A Meta-analysis and Meta-regression. Sports Med..

[37] Patel Y.C. (1999). Somatostatin and Its Receptor Family. Front. Neuroendocr..

[38] Mansell T., Ponsonby A.-L., Collier F., Burgner D., Vuillermin P., Lange K., Ryan J., Saffery R., Barwon Infant Study Investigator Team (2019). Genetic variation, intrauterine growth, and adverse pregnancy conditions predict leptin gene DNA methylation in blood at birth and 12 months of age. Int. J. Obes..

